# Comparison of Early T-Cell Precursor and Non-ETP Subtypes Among 122 Chinese Adults With Acute Lymphoblastic Leukemia

**DOI:** 10.3389/fonc.2020.01423

**Published:** 2020-08-21

**Authors:** Yi Zhang, Jie-Jing Qian, Yi-Le Zhou, Xin Huang, Jian-Hu Li, Xue-Ying Li, Chen-Ying Li, Huan-Ping Wang, Yin-Jun Lou, Hai-Tao Meng, Wen-Juan Yu, Hong-Yan Tong, Jie Jin, Hong-Hu Zhu

**Affiliations:** ^1^Department of Hematology, The First Affiliated Hospital, College of Medicine, Zhejiang University, Hangzhou, China; ^2^Institute of Hematology, Zhejiang University, Hangzhou, China; ^3^Zhejiang Province Key Laboratory of Hematology Oncology Diagnosis and Treatment, Hangzhou, China

**Keywords:** leukemia, T lymphocyte, acute, early T-cell precursors, prognosis, adult, Chinese

## Abstract

**Background:** Adult T-cell acute lymphoblastic leukemia (T-ALL) is a rare hematological malignancy and significantly linked to poor outcomes. Early T-cell precursor (ETP) leukemia is a unique subtype of T-ALL. The aim of this study is to compare the differences between ETP and non-ETP ALLs in China.

**Methods:** We retrospectively analyzed the records of 122 adult T-ALL patients diagnosed and treated at our center between January 2014 and June 2019. All the patients enrolled were categorized into ETP and non-ETP ALL by immunophenotype, and further statistical analyses about clinical data and prognostic factors were performed.

**Results:** Among the 122 cases, the male-to-female ratio was 2.8:1, and the median age is 29 (range, 16–82) years. Except for 10 patients with insufficient immunophenotyping results, 47.3% (53/112) are ETP and 52.7% (59/112) are non-ETP. Compared with non-ETP patients, ETP-ALL patients had lower white blood cell counts and lactate dehydrogenase levels, while they were older and had higher platelet counts and fibrinogen levels (all *p* < 0.05). Complete remission (CR) was achieved in 68.0% (83/122) of patients, 64.2 and 76.3% in ETP and non-ETP, respectively (*p* = 0.160). In total, 44.6% (37/83) of patients relapsed. Allogeneic stem cell transplantation (allo-SCT) was successfully performed in 36.1% (44/122) of patients, of which 79.5% (35/44) were in CR1. With a median follow-up of 9.1 (range, 0.5–70.3) months, the estimated 2-year overall survival (OS) and relapse-free survival (RFS) rates for the cohort were 38.0 ± 5.1 and 39.1 ± 6.3%, respectively. In the ETP group, the 2-year OS rate was 40.7 ± 8.2% and the RFS rate was 47.2 ± 10.7%, while in the non-ETP group, the 2-year OS rate was 37.9 ± 7.0% and the RFS rate was 39.2 ± 8.3% (both *p* > 0.05). In the landmark analysis of CR1 patients who had a survival of more than 6 months, the allo-SCT group had significantly better survival outcomes than the chemotherapy group, and the 2-year OS rates and RFS rates were 80.1 ± 7.3 vs. 28.4 ± 8.4% and 68.9 ± 8.8 vs. 12.8 ± 7.2%, respectively (both *p* < 0.0001). A multivariate analysis suggests that allo-SCT acts as an independent prognostic factor for both OS and RFS.

**Conclusions:** Our results revealed that ETP accounted for a high proportion of T-ALL in Chinese. There are no CR rates and prognosis differences between ETP and non-ETP. Allo-SCT in CR1 can significantly improve patients' survival.

## Introduction

T-cell acute lymphoblastic leukemia (T-ALL) is a malignant clonal disease of the lymphatic system, showing highly heterogeneous immunophenotypes and responses to therapies (1, 2). It accounts for 25% of adult ALL and has inferior outcomes. Based on the corresponding stage of intrathymic differentiation, T-ALLs are divided into pro-T, pre-T/immature, cortical T, and mature-T (3). Currently, risk-based multi-agent chemotherapy and timely bone marrow transplantation are recommended for the treatment of T-ALL.

Early T-cell precursor (ETP) ALL is a gradually recognized T-ALL subtype accounting for ~15% of T-ALL cases in children and 10–30% in adults (4–9). Compared with non-ETP, the ETP patients were older and had lower white blood cell (WBC) counts. Some studies also observed higher platelet (PLT) counts, higher central nervous system (CNS) involvement, and a lower frequency of mediastinal mass in ETP patients (5, 9–11). The initial study of children ETP-ALL described the unfavorable outcomes of this certain subtype (4), and some other studies with small cohorts showed similar results (7, 12). However, some recent reports with large cohorts have shown that children ETP ALL can obtain similar outcomes to non-ETP ALL with reasonable and effective treatments (6, 13). Data on the characteristics and the prognosis of adult ETP ALL were insufficient (14, 15).

Up to now, adult T-ALL is still regarded as a difficult medical problem. Due to the lack of complete Chinese T-ALL data and the insufficient knowledge of the differences between ETP and non-ETP, we aim to compare the characteristics and the prognostic differences between ETP and non-ETP subtypes, which may help to clarify the features and the prognosis of adult T-ALL in Chinese.

## Materials and Methods

### Patients and Classification

**Figure 1:**
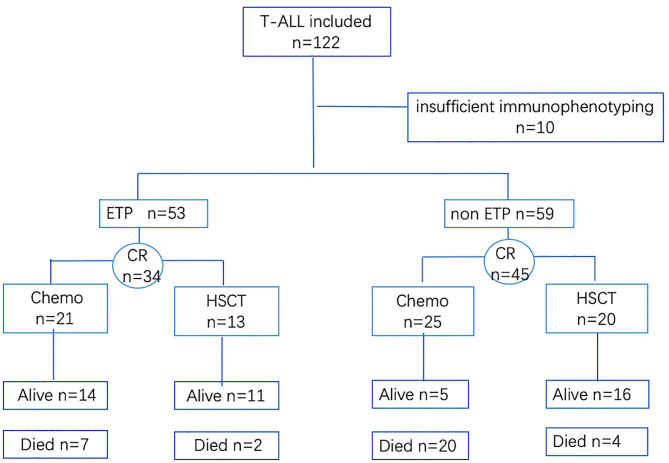
Flow diagram of patient recruitment.

Between January 2014 and June 2019, 122 *de novo* T-ALL patients, 16 years or older of age, were included in this retrospective study. Patients with insufficient medical data or who refused to treatment were excluded (Figure 1). In our hospital, T-lymphoblastic lymphoma (T-LBL) patients were treated at the oncology department instead of at the hematology department, with different treatment regimens; so, the T-LBL patients were excluded in our study. The classification and the diagnostic criteria were based on the 2016 revision to the World Health Organization classification of myeloid neoplasms and acute leukemia (16). The study followed the Declaration of Helsinki and was approved by the Research Ethics Committee of the First Affiliated Hospital, College of Medicine, Zhejiang University.

### Immunophenotyping

A total of 122 patients had immunophenotyping studies in the Laboratory of the First Affiliated Hospital, College of Medicine, Zhejiang University, and designated as T-lineage ALL. ETP was defined as the positive expression of CD7, a lack of CD1a and CD8, weak expression of CD5 (with <75% positive blasts), and positive expression of one or more stem cell or myeloid markers including CD117, HLADR, CD13, CD33, CD11b, or CD65 (4).

### Cytogenetic Analysis

A R-banding chromosome study was performed on diagnostic bone marrow after 24-h culture in RPMI 1640 (Sigma, Schnelldorf, Germany) supplemented with 20% fetal bovine serum, and the chromosome R-banding was analyzed in a minimum of 20 metaphases. Karyotyping findings were described in accordance with the International System for Human Cytogenetic Nomenclature (ISCN, 2013) (17).

### Therapy

Patients who were fit for intensive chemotherapy either received CALLG2008 protocol (a protocol developed by the Chinese Acute Lymphoblastic Leukemia Cooperative Group for ALL) (18) (*n* = 78, 64.0%) or augmented MDACC Hyper-CVAD protocol (19) (*n* = 27, 22.1%) as induction, consolidation, maintenance, and CNS prophylaxis. Patients who were unfit for intensive chemotherapy received dose-reduced chemotherapy regimens (*n* = 17,13.9%). The details of the protocols of CALLG2008 and augmented MDACC Hyper-CVAD for ph-negative ALL are summarized in Supplementary Tables 1, 2.

After achieving complete remission (CR), allo-SCT was recommended to all eligible patients, especially with one of the following risks: high WBC counts (WBC ≥ 100 × 109/L) at diagnosis, high-risk cytogenetics, ≥35 years of age, and positive minimal residual disease detected by flow cytometry at the end of induction. Hematopoietic stem cell transplantation performed at our center included matched sibling donors, unrelated donors, and haploidentical related donors. Conditioning regimens and graft vs. host disease prophylaxis for allo-SCT were reported previously (20).

Patients who did not receive allo-SCT continued with consolidation and maintenance chemotherapy for 2–3 years. The salvage strategies in relapsed patients were based on the discretion of the investigators and the patients.

### End Points

The measured outcomes were CR, overall survival (OS), and relapse-free survival (RFS). CR was defined as <5% bone marrow blasts and showing normal maturation of all cell lineages, no blast in blood, absolute neutrophil count ≥1.0 × 10^9^ /L, platelet count ≥100 × 10^9^ /L, and no extramedullary leukemia. Relapse was defined as a reappearance of blasts in bone marrow (>5%) or extramedullary leukemia in patients with previously documented CR. OS was defined as the time from the first treatment to death or last follow-up. RFS was defined as the time from first CR to relapse, censoring at death in CR or last follow-up. The last follow-up time was November 2019, and 9.0% (11/122) were lost to follow-up.

### Statistical Analysis

Continuous variables were analyzed by Mann–Whitney *U*-test and the categorical parameters were compared by Pearson χ^2^ test or Fisher's exact test. OS and EFS probabilities were estimated using the Kaplan–Meier survival analysis, and the differences in survival curves were compared by log-rank test. The standard binary logistic regression model was applied in CR univariates and multivariate analysis. Multivariate Cox hazard models were used to estimate the hazard ratio for OS and RFS. The landmark was chosen as a median interval from first chemotherapy to transplant: 6 months. The univariate analyses included sex (male vs. female), age (<35 vs. ≥35 years), white blood cells (<100 vs. ≥100 × 10^9^/L), hemoglobin (<100 vs. ≥100 g/L), platelet (<35 vs. ≥35 × 10^9^/L), lactate dehydrogenase (LDH; <245 vs. ≥245 U/L), fibrinogen (<3.15 vs. ≥3.15 g/L), diabetes mellitus (yes/no), bone marrow blasts (<60 vs. ≥60%), ETP (yes/no), and allo-SCT (yes/no). Factors with *P* < 0.2 were used in the multivariate analyses. All statistical analyses were performed using SPSS Version 25. *P* < 0.05 were considered as significant.

## Results

### Patients' Characteristics

A total of 122 patients were included, and 73.8% (90/122) were male, with a male-to-female ratio of 2.8:1. The median age was 29 (range, 16–82), and 15.6% were over 60 years old. Informative karyotypic results were obtained in 81.1% (99/122) patients, and abnormal karyotypes were found in 27.3% (27/99) cases, including 9.1% (9/99) complex karyotype (≥5 abnormalities). Within this cohort, 112 patients had adequate immunophenotype data, and 47.3% (53/112) of cases were classified into ETP, with the remaining 52.7% (59/112) classified into non-ETP.

The clinical characteristics of ETP and non-ETP were compared, and detailed data are shown in Table 1. The ETP cases were older than the non-ETP cases, with the median age of 38 (16–73) and 23 (16–67) years, respectively (*P* < 0.001). Compared with non-ETP, ETP patients had lower WBC counts and lower LDH levels but higher PLT counts and fibrinogen levels (all *p* < 0.05).

**Table 1:** Characteristics of early T-cell precursor acute lymphoblastic leukemia (ETP-ALL) and non-ETP-ALL patients.

**Variable**	**T-ALL (*n* = 122)**	**ETP (*n* = 53)**	**Non-ETP (*n* = 59)**	***P***
Sex				0.231
Male	73.8	67.9	78.0	
Female	26.2	32.1	22.0	
Age, years	29 (16–82)	38 (16–73)	23 (16–67)	
≥35 years old	36.9	52.8	18.6	**0.000**
White blood cell, 10^9^/L	12.2 (0.3–817.7)	7.5 (0.3–117.9)	28 (1.1–817.7)	
≥100 × 10^9^/L	17.2	3.8	28.8	**0.000**
Hemoglobin, g/L	95 (42–176)	87 (51–156)	107 (42–176)	0.122
Platelet, 10^9^/L	79 (4–436)	110 (20–436)	61 (4–321)	**0.001**
LDH, U/L	370 (141–22,260)	339 (141–1,347)	461 (143–7,349)	**0.023**
>245, U/L	73.0	66.0	75.9	0.264
Fibrinogen, g/L	2.79 (0.27–6.8)	3.37 (1.08–6.8)	2.38 (0.27–5.95)	**0.001**
D-dimer, μg/L	1,801 (6–51,860)	1,520 (180–41,860)	2,078 (6–51,860)	0.063
DM (FBS > 7.0 mmol/l)	14.5	18.5	10.7	0.267
Mediastinal mass	28.5	22.6	33.9	0.188
BM blasts	78 (20–97)	80 (20–95)	77.5 (20–97)	0.461
Karyotype*				0.554
Normal karyotype	77.8	78.0	68.0	
Complex karyotype	9.1	7.3	12.0	
Others	13.1	14.7	20.0	
Induction therapy				0.614
CALLG2008	63.9	64.2	71.8	
Hyper CVAD	22.1	26.4	18.6	
Others	13.9	9.4	10.2	
CR rate	68.0	64.2	76.3	0.160
Allo-SCT				0.444
In all	36.1	32.0	42.4	
In CR1	27.9	24.5	35.6	

The values are median (ranges) or percentage. The significant P-values are in bold.

Karyotype ^*^ complex karyotype ≥5 anomalies.

*ETP, early T-cell precursor; LDH, lactate dehydrogenase; DM, diabetes mellitus; FBS, fasting blood sugar; BM, bone marrow; CALLG2008, regimen of vincristine, daunorubicin, cyclophosphamide, and prednisone with or without asparaginase or daunorubicin was replaced by idarubicin; Hyper CVAD, hyper-CVAD A regimen of cyclophosphamide, vincristine, adriamycin, and dexamethasone; Others, low-dose induction therapy regimen; CR, complete remission; Allo-SCT, allogeneic stem cell transplantation*.

Overall, allo-SCT was performed in 36.1% (44/122) of patients, including 81.8% (35/44) in CR1, with a median interval from first chemotherapy to transplant of 5.9 (range, 4.1–16) months. The main demographic and the clinical data of allo-SCT and non-allo-SCT are listed in Table 2.

**Table 2:** Comparison of the characteristics of patients with allo-hematopoietic stem cell transplantation (allo-HSCT) or without HSCT.

**Variable**	**Allo-HSCT (*n* = 44)**	**Without HSCT (*n* = 80)**	***P***
Sex			0.276
Male	79.5	70.5	
Female	20.5	29.5	
Age, years	23 (16–50)	42 (16–82)	
≥35 years old	6.8	53.8	0.000
White blood cell, 10^9^/L	13.8 (1.3–234.6)	11.9 (0.3–817.7)	
≥100 × 10^9^/L	15.9	17.9	0.774
Hemoglobin, g/L	103.0 (42–166)	93 (53–176)	0.801
Platelet, 10^9^/L	97 (4–436)	73.5 (13–319)	0.127
LDH, U/L	335.5 (144–6,073)	141 (409–22,260)	0.155
>245, U/L	60.0	80.0	0.021
Fibrinogen g/L	2.7 (0.27–6.80)	2.96 (0.86–6.64)	0.359
D-dimer μg/L	1,856 (160–21,100)	1,666 (6–51,860)	0.785
DM (FBS > 7.0 mmol/l)	15.0	14.3	0.917
BM blasts	77 (20–94)	78.5 (27–97)	0.681
Induction therapy			0.505
CALLG2008	65.9	61.3	
Hyper CVAD	27.3	18.7	
Others	6.8	20.0	

*The values are median (ranges) or percentage*.

### Outcome

All T-ALL cases received chemotherapy as induction therapy, including 64.0% (77/122) with VDCP-based regimens, 22.1% (27/122) with hyper-CVAD A, and 13.9% (17/122) with dose-reduced chemotherapy regimens. Complete remission was obtained in 68.0% (83/122) of patients. A total of 4.9% (6/122) patients died during the first induction therapy; slightly fewer patients with ETP phenotype achieved CR compared with those with non-ETP (64.2 vs. 76.3%, *p* = 0.160). Relapse occurred in 44.6% (37/83) of patients, including eight patients who relapsed after transplantation.

**Figure 2:**
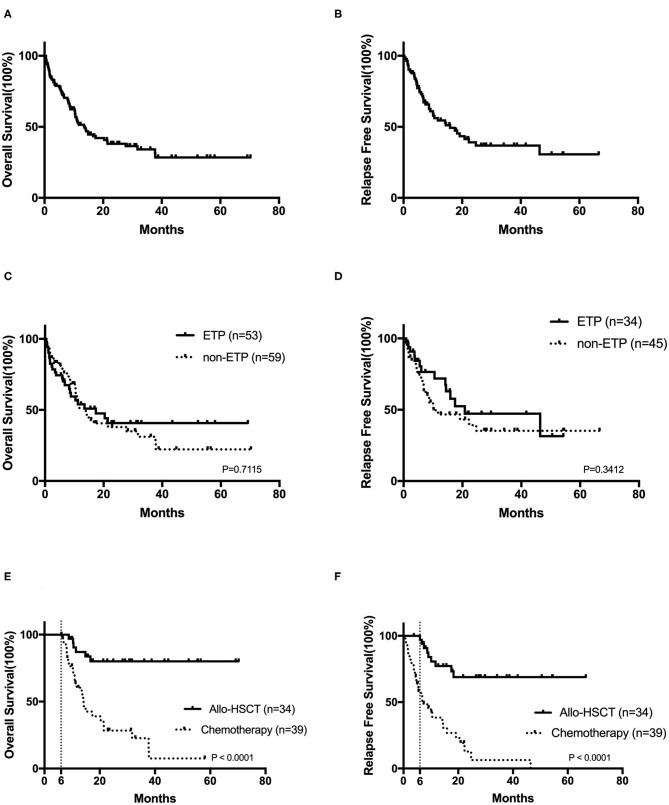
The prognosis of T-cell acute lymphoblastic leukemia. Kaplan–Meier analyses for overall survival **(A)** and relapse-free survival **(B)** for all 122 patients. Overall survival **(C)** and relapse-free survival **(D)** in early T-cell precursor acute lymphoblastic leukemia (ETP-ALL) and non-ETP-ALL. The overall survival **(E)** and the relapse-free survival **(F)** in CR1 patients by landmark analysis, with a landmark point at 6 months.

After a median follow-up of 9.1 (range, 0.5–70.3) months, the 2-year OS and RFS rates were 38.0 ± 5.1 and 39.1 ± 6.3%, respectively (Figures 2A,B). No survival differences were found in ETP and non-ETP (ETP vs. non-ETP: 2-year OS rate 40.7 ± 8.2 vs. 37.9 ± 7.0%, *p* = 0.712; 2-year RFS rate 47.2 ± 10.7 vs. 39.2 ± 8.3%, *p* = 0.341; Figures 2C,D), even with the censored allo-SCT time (*p* = 0.751 for OS, *p* = 0.264 for RFS). When the patients were separated into CR1 allo-SCT group and chemotherapy group, there were still no differences in OS between ETP and non-ETP (CR1 allo-SCT group ETP vs. non-ETP and chemotherapy group ETP vs. non-ETP, *p* = 0.258 and 0.717, respectively).

In order to analyze the impact of transplantation on prognosis, we also compared the outcomes in CR1 patients by landmark analysis, with a landmark point at 6 months. Our results suggested that bone marrow transplantation significantly improves patients' survival (Figures 2E,F). The 2-year OS and RFS rates of CR1 patients who had a survival of more than 6 months were 80.1 ± 7.3 and 68.9 ± 8.8% in the allo-SCT group and 28.4 ± 8.4 and 12.8 ± 7.2% in the chemotherapy group (both *p* < 0.0001).

### Univariate and Multivariable Analyses

A multivariate analysis showed that CR rate was strongly affected by hemoglobin (<100 g/L) [OR = 0.205 (95% CI: 0.068–0.614); *p* = 0.005] and fibrinogen (≥3.15 g/L) [OR = 0.256 (95% CI: 0.093–0.710); *p* = 0.009]. Patients who had a comorbidity with diabetes mellitus had shorter OS [HR = 2.266 (95% CI: 0.128–4.551); *p* = 0.022]. Patients who received allo-SCT correlated with a better OS [HR = 0.127 (95% CI: 0.065–0.251); *p* = 0.000] and RFS [HR =0.262 (95% CI: 0.133–5.15); *p* = 0.000; Supplementary Table 3].

## Discussion

In this study, we presented the characteristics of 122 adult T-ALL patients and compared the heterogeneity between ETP and non-ETP in detail. To our knowledge, this is a considerable large, single-center data of Chinese adult T-ALL, with a comprehensive analysis of ETP and non-ETP. We found that ETP accounts for a high proportion of T-ALL patients in China. The prognosis of T-ALL in Chinese patients is still poor, and no CR rate and survival differences were found between ETP and non-ETP groups. Allo-SCT in CR1 can significantly improve patients' survival.

ETP occupies a high proportion of Chinese adult T-ALL. It was observed that ETP patients accounted for 45% of all T-ALL in our center, which was consistent with the result of Liao *et al*. from another Chinese center (47.6%) (11), but our data is much higher than that of some international studies, which were 17, 22, and 32%, respectively (5, 9, 21). There may be ethnic differences in the incidence of ETP. Other patient characteristics in our research, like the age of disease onset, gender ratio, and blood routine level of T-ALL and ETP, were similar to those of international investigations.

The long-term outcome of T-ALL in China is dismal, and the overall survival rates remain only 20–40% (10, 11). The CR, 2-year OS, and 2-year RFS rates in our research were 68.0, 38.0 ± 5.1, and 39.1 ± 6.3%, which were lower than those of some high-quality international researches (5, 13, 21). This might be attributed to the higher proportion of elderly patients in our study, where 15.6% patients were over 60 years old. Thus, the outcomes of elderly (>60 years old) vs. non-elderly patients were analyzed. In the non-elderly group, the 2-year OS rates were 42.3 ± 5.5%, the 2-year RFS rates were 43.2 ± 6.5%, and the median OS was 14.2 (95% CI: 5.4–23.1) months, while in the elderly group the median OS was only 6.7 (95% CI 10.2–17.2) months, and all the patients died within 2 years. Besides that, some patients had severe comorbidities, which prevented them from receiving standard doses of induction therapy and eventually led to poor outcomes. In addition, since minimal residual disease (MRD) may be used to allocate patients to different treatments and only a part of the patients in our study underwent the MRD test, such may also lead to a different prognosis compared with other centers.

The survival differences between ETP and non-ETP might still be controversial. In our research, there were no survival differences between these two subtypes, which was in line with the results of the German Multicenter Study Group of Adult ALL (9) and the Group for Research on Adult ALL (GRAALL) (21). However, the studies of the St. Jude Children's Research Hospital (4) and of the University of Texas MD Anderson Cancer Center (MDACC) (5) demonstrated that ETP was highly invasive and had a significantly worse prognosis than other subtypes of T-ALL. In the report of the MDACC study group, the patients were treated with Hyper-CVAD ± nelarabine or augmented Berlin–Frankfurt–Munster regimen, and routine allo-SCT in the first clinical remission (CR1) was not recommended. They proved that ETP-ALL/LBL represented a high-risk disease subtype of adult ALL, with lower CR/CRp rate (73 vs. 91%; *P* = 0.03) and median OS (20 months vs. not reached, *p* = 0.008) than those of non-ETP patients. In the research of GRAALL, the patients were treated in the GRAALL 2003 and 2005 studies; allo-SCT (ETP, 48.9% and non-ETP, 28.3%) was produced in some high-risk patients. They revealed that patients with ETP had outcomes similar to those of the non-ETP cohort, with no significant differences in either the 5-year OS or the EFS rates. The differences may be related to the effective induction therapy regimens of ETP, the percentage of allo-SCT, and the use of MRD. In our study, we found that transplantation was still the critical treatment modality and could significantly improve the prognosis both in the ETP and the non-ETP groups. As to the insufficient knowledge of adult T-ALL, further improvements like therapeutic approaches and risk stratification are still needed (22–24).

However, this study has some limitations despite being a large reported series of adult T-ALL and ETP subtypes. One important limitation is the retrospective and single-center design, which may lead to the heterogeneity of patients and a high lost-to-follow-up rate. These may cause a deviation in the prognostic analysis. Another limitation of the study is that 15.6% of the patients in our study were elderly (>60 years old) and some had severe complications, which could prevent them from receiving intensive chemotherapy, finally leading to poor outcomes.

In summary, ETP accounted for a high proportion of T-ALL in Chinese. There are no significant differences in CR rates and long-term survival between ETP and non-ETP groups. Allo-SCT in CR1 can significantly improve the survival of T-ALL patients. At present, the outcome of T-ALL is still poor, and novel therapies are urgently awaited.

## Data Availability Statement

The raw data supporting the conclusions of this article will be made available by the authors, without undue reservation.

## Ethics Statement

The studies involving human participants were reviewed and approved by the Research Ethics Committee of the First Affiliated Hospital, College of Medicine, Zhejiang University. The patients/participants provided their written informed consent to participate in this study.

## Author Contributions

H-HZ, YZ, and J-JQ drafted the manuscript and contributed to the final draft, and the other authors collected the data for the manuscript. All authors reviewed and approved the final draft, contributed to the article, and approved the submitted version.

## Conflict of Interest

The authors declare that the research was conducted in the absence of any commercial or financial relationships that could be construed as a potential conflict of interest.
