# Supplementary material for: Comparison of Early T-Cell Precursor and Non-ETP Subtypes Among 122 Chinese Adults With Acute Lymphoblastic Leukemia
Source: Front Oncol. 2020 Aug 21;10:1423. doi: 10.3389/fonc.2020.01423 (PMC7473208; doi:10.3389/fonc.2020.01423)
Supplement: Supplementary file 1 [file Data_Sheet_1.docx]

**Supplementary Table 1**

**CALLG2008 chemotherapy protocol**

| **Treatment phase** | **Dose** | **Route** | **Days** |
| --- | --- | --- | --- |
| **Prephase (WBC≥50×109/L)** |  |  |  |
| Prednisone | 60 mg/d | PO | D -3 to 1 |
| Cyclophosphamide | 200 mg/m2 | IV | D -3 to 1 |
| **Frontline induction (4 weeks)** |  |  |  |
| VDCLP (I) |  |  |  |
| Vindesine | 4 mg/d | IV | D1, 8, 15, 22 |
| Daunorubicin* | 40 mg/(m2/d) | IV | D1, 8, 15, 22 |
| Cyclophosphamide | 1 mg/(m2/d) | IV | D1, D15 |
| Asparaginase^#^ | 6000IU/m2/d | IM | D11,14,17, 20, 23, 26 |
| Prednisone | 1 mg/(m2/d) | PO | D1–14, D15–28 (2/3 dose) |
| **Early stage consolidation block** | | | |
| 1. CAM (II) |  |  |  |
| Cyclophosphamide | 750 mg/m2 | IV | D1, 8 |
| Cytarabine | 100 mg/(m2/d) | IV | D1–3, D8–10 |
| 6-Mercaptopurine | 60 mg/(m2/d) | PO | D1–7 |
| 2. HD-MTX + L-ASP(III) |  |  |  |
| Methotrexate | 3-5 g/m2 | IV | D1 |
| Asparaginase^#^ | 6000IU/(m2/d) | IM | D3,4 |
| 3. MA (IV) |  |  |  |
| Mitoxatrone | 8 mg/(m2/d) | IV | D1–3 |
| Cytarabine | 750 mg/m2 q12h | IV | D1–3 |
| **Late stage consolidation block** | | | |
| 1.VDLP(V) |  |  |  |
| Vindesine | 4 mg/d | IV | D1, 8, 15, 22 |
| Daunorubicin* | 40 mg/(m2/d) | IV | D1, 8, 15, 22 |
| Asparaginase^#^ | 6000IU/(m2/d) | IM | D11,14,17,20,23,26 |
| Dexamethasone | 8 mg/(m2/d) | PO | D1–7, D15–21 |
| 2. COATD(VI) |  |  |  |
| Cyclophosphamide | 750 mg/m2 | IV | D1 |
| Vindesine | 4 mg/d | IV | D1 |
| Cytarabine | 100 mg/(m2/d) | IV | D1-7 |
| Teniposide | 100 mg/(m2/d) | IV | D1-4 |
| Dexamethasone | 8 mg/(m2/d) | IV | D1-7 |
| 3. HD-MTX+L-ASP (VII) |  |  |  |
| Methotrexate | 3-5 g/m2 | IV | D1 |
| Asparaginase^#^ | 6000IU/(m2/d) | IM | D3,4 |
| 4. TA(VIII) |  |  |  |
| Teniposide | 100 mg/(m2/d) | IV | D1-4 |
| Cytarabine | 100 mg/(m2/d) | IV | D1-7 |
| **Long-term maintenance (until 36 months from diagnosis)** | | | |
| Methotrexate | 20 mg/(m2/week) | PO | Day 8 of every 4 weeks |
| 6-Mercaptopurine | 60 mg/(m2/d) | PO | D1–7 of every 4 weeks |
| **consolidation block (every 6 months for high risk patients)** | | | |
| MOACD |  |  |  |
| Mitoxatrone | 8 mg/(m2/d) | IV | D1–2 |
| Vindesine | 4 mg/d | IV | D1 |
| Cyclophosphamide | 600 mg/m2 | IV | D1 |
| Cytarabine | 100 mg/(m2/d) | IV | D1-5 |
| Dexamethasone | 6 mg/(m2/d) | IV | D1-7 |

Abbreviations: IV, intravenous; IM, intramuscular; PO, oral; *Or idarubicin 8 mg/(m2/d). ^#^ some patients received Asparaginase. Patients received intrathecal chemotherapy treatments administered during the consolidation courses as a prophylactic for relapse in the CNS. Intrathecal (IT) chemotherapy: cytarabine (50 mg) and/or methotrexate (10 mg), and dexamethasone (5 mg).

**Supplementary Table 2**

**Augmented M.D. Anderson Hyper-CVAD protocol**

| **Treatment phase** | **Dose** | **Route** | **Days** |
| --- | --- | --- | --- |
| **Dose-intensive phase*** | | | |
| 1. Hyper-CVAD A |  |  |  |
| Cyclophosphamide | 300 mg/(m2/q12h) | IV | D1-3 |
| Vindesine | 4 mg/d | IV | D4,11 |
| Doxorubicin | 50 mg/m2 | IV | D4 |
| Dexamethasone | 40mg/d | IV | D1-4, D11-14 |
| 2. Hyper-CVAD B |  |  |  |
| Methotrexate | 1 g/m2 | IV | D1 |
| Cytarabine^#^ | 2 g/(m2/q12h) | IV | D2-3 |
| **Maintenance phase (for 2 years)** | | | |
| POMP^ |  |  |  |
| 6-Mercaptopurine | 60 mg/(m2/d) | PO | D1–7 |
| Methotrexate | 20 mg/(m2/week) | PO | weekly |
| Vindesine | 4 mg/d | IV | D1 |
| Prednisone | 100mg | PO | D1-5 |

*Patients received eight induction-consolidation cycles of hyper-CVAD A (cycles 1, 3, 5, 7) alternating with Hyper-CVAD B (cycles 2, 4, 6, 8) every 28-35 days; ^#^the dose of cytarabine was reduced from 3 g/(m2/q12h) to 2 g/(m2/q12h); ^the dose of 6-Mercaptopurine and prednisone were reduced.

**Supplementary Table 3**

**Univariate and multivariate analysis for CR and survival**

| Parameter | Complete remission | | | Overall survival | | | Relapse-free survival | | |
| --- | --- | --- | --- | --- | --- | --- | --- | --- | --- |
|  | Univariate | Multivariate | | Univariate | Multivariate | | Univariate | Multivariate | |
|  | P value | OR (95% CI) | P value | P value | HR (95% CI) | P value | P value | HR (95% CI) | P value |
| Sex (male vs. female) | 0.224 | - | - | 0.843 | - | - | 0.709 | - | - |
| Age ≥35 years | **0.000** | 0.476(0.158-1.437) | 0.188 | **0.000** | 1.260(0.725-2.188) | 0.412 | 0.015 | 1.075(0.509-2.269) | 0.850 |
| White blood cell ≥100 × 109/L | 0.883 | - | - | 0.347 | - | - | 0.734 | - | - |
| Hemoglobin <100 g/l | **0.026** | 0.205(0.068-0.614) | **0.005** | 0.632 | - | - | 0.928 | - | - |
| Platelet <35× 109/L | 0.216 | - | - | 0.121 | 1.247(0.707 -2.198) | 0.446 | 0.109 | 1.492(0.739-3.031) | 0.264 |
| LDH >245U/L | 0.099 | 0.379(0.118-1.214) | 0.102 | 0.058 | 0.908(0.453-1.818) | 0.785 | 0.151 | 1.244(0.572-2.707) | 0.582 |
| Fibrinogen ≥3.15g/L | **0.003** | 0.256(0.093-0.710) | **0.009** | **0.022** | 1.653(0.978-2.792) | 0.06 | 0.651 | - | - |
| Diabetes mellitus (yes/no) | 0.070 | 0.445(0.100-1.968) | 0.286 | **0.016** | 2.266(1.128-4.551) | **0.022** | 0.640 | - | - |
| BM blasts ≥60% | 0.787 | - | - | 0.920 | - | - | 0.837 | - | - |
| ETP (yes/no) | 0.162 | 1.229(0.394-3.839) | 0.722 | 0.712 | - | - | 0.344 | - | - |
| Allo-SCT (yes / no) | - | - | - | **0.000** | 0.127(0.065-0.251) | **0.000** | 0.000 | 0.262(0.133-5.15) | **0.000** |

Factors (univariate analysis p<0.2) underwent multivariate analysis. Significant P values are in bold.

Abbreviations: OR, odds ratio; HR, hazard ratio; LDH: lactate dehydrogenase; BM: bone marrow; Allo-SCT: allogeneic stem cell transpla
